# Cancer immunotherapy and its facilitation by nanomedicine

**DOI:** 10.1186/s40364-024-00625-6

**Published:** 2024-08-03

**Authors:** Chao Sui, Heqing Wu, Xinxin Li, Yuhang Wang, Jiaqi Wei, Jianhua Yu, Xiaojin Wu

**Affiliations:** 1 Department of Hematology & Hematopoietic Cell Transplantation, City of Hope National Medical Center, 1500 East Duarte, Los Angeles, CA 91010 USA; 2 The First Affiliated Hospital of Soochow University, Suzhou, China; 3 National Clinical Research Center for Hematologic Diseases, Jiangsu Institute of Hematology, Suzhou, China; 4 Institute of Blood and Marrow Transplantation, Collaborative Innovation Center of Hematology, Soochow University, Suzhou, China; 5 Xi’an Key Laboratory of Stem Cell and Regenerative Medicine, Institute of Medical Research, Northwestern Polytechnical University, Xi’an Shaanxi, 710072 China; 6 Hematologic Malignancies Research Institute, City of Hope National Medical Center, Los Angeles, CA 91010 USA

**Keywords:** Cancer, Immunotherapy, Nanomedicine, Nanotechnology

## Abstract

Cancer immunotherapy has sparked a wave of cancer research, driven by recent successful proof-of-concept clinical trials. However, barriers are emerging during its rapid development, including broad adverse effects, a lack of reliable biomarkers, tumor relapses, and drug resistance. Integration of nanomedicine may ameliorate current cancer immunotherapy. Ultra-large surface-to-volume ratio, extremely small size, and easy modification surface of nanoparticles enable them to selectively detect cells and kill cancer cells in vivo. Exciting synergistic applications of the two approaches have emerged in treating various cancers at the intersection of cancer immunotherapy and cancer nanomedicine, indicating the potential that the combination of these two therapeutic modalities can lead to new paradigms in the treatment of cancer. This review discusses the status of current immunotherapy and explores the possible opportunities that the nanomedicine platform can make cancer immunotherapy more powerful and precise by synergizing the two approaches.

## Introduction

Despite significant efforts to develop quality therapies aimed at eliminating cancer, it still remains the second leading cause of death worldwide over the past decades [1, 2]. Clinical data suggest two major challenges to be overcome in cancer therapy: (1) the metastasis or spread of cancer cells [3, 4] and (2) the recurrence of dormant cells [5]. It has been recognized that immune cells play a pivotal role in controlling tumor growth, invasion, and metastasis [6, 7]. Importantly, emerging evidence suggests that each step of tumor development, from initiation through metastatic spread, involves the communication between tumor and immune cells via the secretion of cytokines and growth factors [8, 9].

**Fig. 1:**
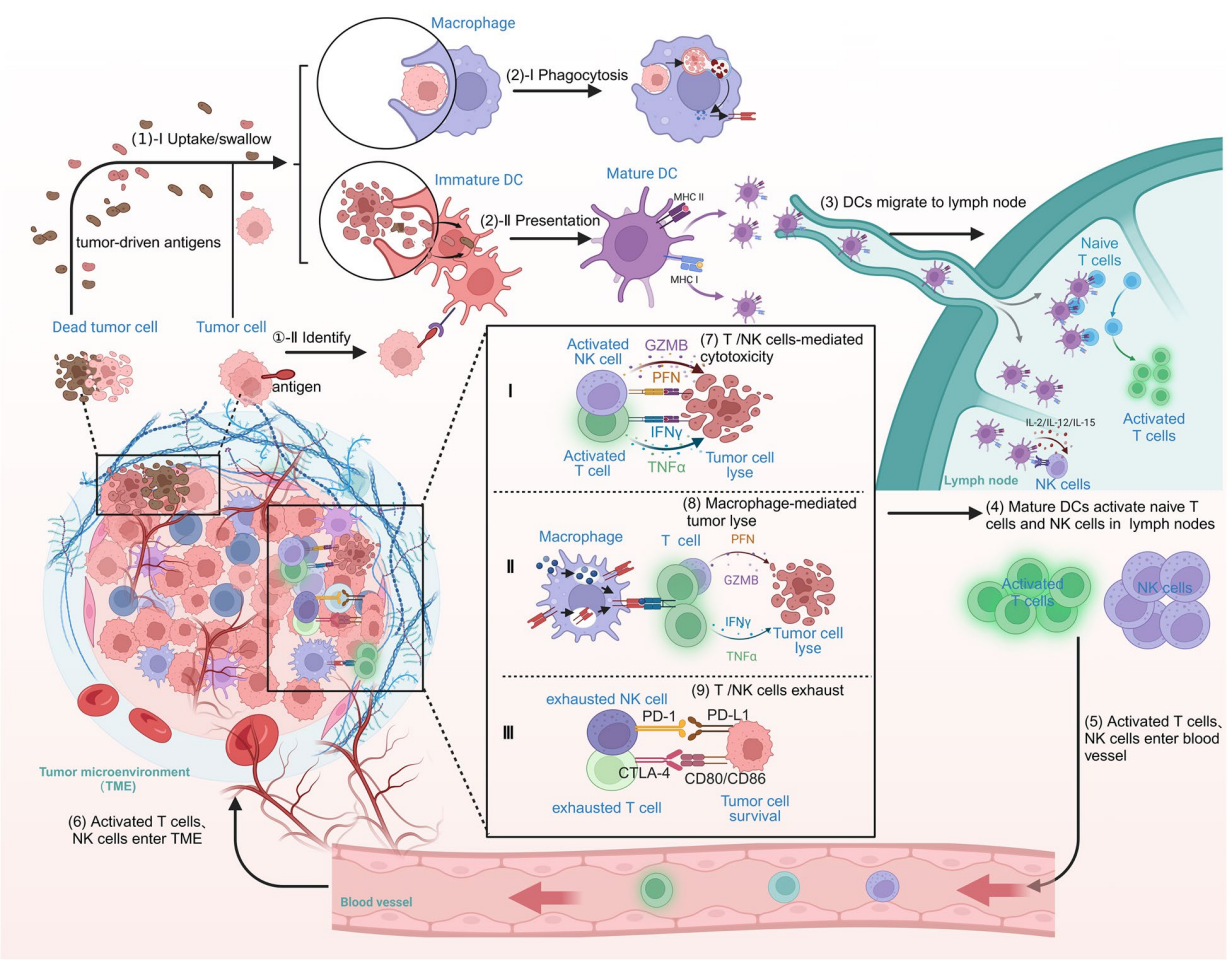
Schematic of generation and regulation of the anti-tumor immunity. The response begins with the capture of tumor-driven antigens by DCs or macrophages, and then, DCs present antigens through MHC molecules. Next, the mature DCs will migrate to the lymph nodes through lymphatic vessels, which can activate the naive T cells and NK cells. These antigen-educated T and NK cells exit the lymph node and enter the tumor to perform their functionhttp://www.BioRender.com

To combat malignant cancer, it is undoubtedly that the immune system plays an important role. The immune system can be broadly divided into two categories: (1) the innate immune system, mainly composed of epithelial barriers, mucous membrane, monocytes and macrophages, granulocytes, neutrophils, dendritic cells (DCs), and natural killer (NK) cells [10]; and (2) the adaptive immune system, whose main components are humoral immunity dominated by B cells and cellular immunity dominated by T cells [10, 11]. In the tumor microenvironment (TME), activation of effective anti-tumor immunity requires the simultaneous engagement of the innate and adaptive immune systems. The specific execution processes summarized in Fig. 1, briefly: (1)-(2) Immature DCs phagocytose tumor-derived antigens (Ags) or recognize antigens present on tumor cells, subsequently inducing their maturation. In addition, macrophages directly engulf tumor cells via phagocytosis [12]; (3)-(4) mature DCs migrate to the lymph nodes through lymphatic vessels to active naïve T and NK cells [13, 14], (5)-(6) activated T and NK cells enter the TME through blood vessel [15, 16]; (7)-(9) in the TME, activated T and NK cells or macrophages mediated tumor lyse. However, in the TME, NK and T cells will be gradually exhausted, and exhausted T and NK cells promote tumor immune escape [17, 18].

Cancer immunotherapy, as a biological therapy, is a type of treatment that mainly takes advantages of the artificially stimulated innate and adaptive immune system to treat cancer by affecting immune cells instead of cancer cells in the above execution processes [19]. After years of developments since the first study by William Coley in 1891, various hematological and solid tumors can be treated by cancer immunotherapeutics [20, 21]. However, cancer immunotherapy still faces substantial challenges. For instance, mechanistic insights (molecular or cellular) into regulating genetic mutations, the infiltration of immune cells into the tumor bed, and the formation of immune tolerance remain elusive [22–24]. The treatment effects differ from patient-to-patient and cancer-to-cancer [25]. Devastatingly, it’s difficult to effectively and safely control the immune response following stimulation [26]. Moreover, the treatment expenses of current primary cancer immunotherapy can be unaffordable for most cancer patients. For example, CAR-T cell therapy is very expensive, it may cost up to $500,000 USD for a patient per year [27, 28].

To overcome these barriers, nanotechnology is being applied in the field of cancer immunotherapy by its physical (e.g., size, shape, and mechanical) and surface properties. The technology has the potential to improve the possibility of designing intelligent agents that can selectively detect cells and kill cancer cells in vivo [29, 30]. Nanomedicine, as defined by the National Institutes of Health (NIH), constitutes a branch of nanotechnology that pertains to exceedingly precise medical interventions at the molecular scale, aimed at curing diseases or restoring damaged tissues. This research field has been largely driven over the past decades and now emerges as a powerful platform for cancer treatment, particularly for cancer imaging, diagnostic, and theragnostic [31–33].

In this review, with the context of immuno-oncology, we elaborate on how cancer nanomedicine, an intersection field of cancer nanotechnology and cancer therapy, is revolutionizing cancer immunotherapy. The first objective is to make a brief overview of the current representative cancer immunotherapy. The second objective is to introduce the development of the cancer nanomedicine platform and how it revalorizes current cancer immunotherapy. The third objective is to provide knowledge of current clinical stage of nanomedicine for cancer immunotherapy. The final objective is to summarize the current challenges of clinical translation.

## Current cancer immunotherapy

Cancer cells have developed escape mechanisms by which they co-opt both innate and adaptive immune cells to defect host immune surveillance for tumor progression. As mentioned in Fig. 1, several distinct steps must be achieved to mount effective anti-tumor immunity. Additionally, cytokines, chemokines, and the associated pathways are the main intermediaries involved in the crosstalk between cancer cells and host cells, which underlies the adjustment of tumor evasion. Thus, it’s necessary to focus on the exploration of their mechanisms to generate and regulate anti-tumor immunity [34, 35]. According to the descriptions above, we can interevent tumor evasion from at least three aspects by using various cytokines, chemokines, small molecule drugs, antibodies/proteins, viruses, or even cells [36–38], promoting the antigen presentation functions, promoting the production of active immune cells, and overcoming immune suppression in the tumor bed [19, 39].

### Representative cancer immunotherapeutics

Representative immunotherapeutics can be summarized as: (1) Cancer vaccines. Cancer vaccines, designed to boost immune cells [40, 41], can be further subdivided into bacterial- and viral vector-based vaccines [42, 43], peptide and protein-based vaccines [44], cellular (whole cell and DC)-based vaccines [45], and nucleic acid-based vaccines including RNA vaccines, DNA vaccines, mRNA vaccines, and self-amplifying RNAs (saRNA) vaccines [46–48]. (2) Adoptive cell therapies (ACTs). ACTs involve the process of expanding the patient’s own T cells, NK cells or other cells with or without engineering, followed by infusion of the expanded cells into the patient with cancer [49, 50]. T-cell based cell therapy is the most popular ACT. T cell-based ACTs can be executed through at least three distinct T-cell methodologies. The first one is tumor-infiltrating lymphocytes (TILs)-based ACT, in which endogenous TILs are expanded ex vivo from a patient’s tumor before being infused back into the patient [50, 51].The second one is engineered T-cell receptor (TCR)-based ACT to recognize specific tumor antigens while it’s limited to major histocompatibility complex (MHC) expressing Ags [51]. The third one is chimeric antigen receptor (CAR)-based ACT, in which T cells from patients are engineered with a chimeric receptor consisting of an extracellular antigen recognition domain, a transmembrane domain, and a cytoplasmic signaling domain, making CAR-T cells lock onto and destroy the exact kinds of cancer [52]. (3) Immune checkpoint inhibitors (ICIs). ICIs are drugs that re-activate anti-tumor immunity by blocking co-inhibitory signaling pathways and promoting immune-mediated tumor cell clearance processes [53, 54]. For example, to evade immune surveillance, some cancer cells overexpress the programmed death-ligand 1 (PD-L1) on their surface. This will turn the “cytotoxicity brake” function on, resulting in the exhaustion of T cells and survival of the PD-L1 positive cancer cells [55]. Besides programmed cell death protein 1 (PD-1) and its ligand (PD-L1), the proteins cytotoxic T-lymphocyte-associated antigen 4 (CTLA-4) [56], lymphocyte activation gene 3 (LAG-3) [57, 58], and T-cell immunoglobulin mucin-3 (TIM-3) [59] are also common immune checkpoints (ICPs). (4) Monoclonal antibodies (mAbs). mAbs are large glycoproteins produced by B cells, targeting tumor cells directly while simultaneously promoting the induction of long-lasting anti-tumor immune responses [60]. Three types of mAbs are widely used to treat cancer, naked (or unconjugated) mAbs with nothing attached [61], conjugated mAbs with a chemotherapy drug or radioactive particle attached which are termed antibody–drug conjugates (ADCs) [62, 63], and bispecific mAbs with two different proteins attached at the same time [64]. Importantly, based on the type of heavy chain structure, there are many types of mABs and IgG is the most often form used in antibody therapy currently [65]. Ab scaffold plays an important role in tumor immunotherapy. A recent study showed that rabbit-derived Ab scaffold, a VL single domain antibody scaffold, can effectively connect 7-ethyl-10-hydroxycamptothecin (SN-38) drugs, and significantly inhibit the in vitro and in vivo proliferation of canine non-Hodgkin lymphoma (cNHL) cells, providing valuable theoretical support for Ab scaffold in tumor immunotherapy [66]. (5) Oncolytic virus (OVs) therapy. As one of the frontiers for cancer immunotherapy [67], OVs have a therapeutic potential due to their function of selectively replicating in and killing cancer cells, and spreading within the tumor, while not harming normal tissue [68, 69]. Moreover, it can be treated as an in situ vaccine [70] while it can be uploaded with immune modulatory transgenes [71–75], or even can be combined with other immunotherapeutics, such as cell therapy and chemotherapy [76, 77].

### Pros and cons of cancer immunotherapy

Compared to traditional cancer treatments, surgical intervention, radiation therapy, and chemotherapy, immunotherapy drives significant effectiveness at prolonging the survival of patients with multiple steps, targets, and directions in attacking cancer cells, because immunotherapy can selectively target tumor tissues and reduce the damage of normal tissues [78, 79]. Cancer cells can establish mechanisms to escape immune surveillance, leading to metastases and recurrence, which are two vital factors causing death [3, 80, 81]. Immunotherapy is unequivocal through various methods to stimulate innate immunity and adaptive immunity, remodel the immune suppression in the TME to better tackle the targeted cancer cells, and amplify the pre-existing immunity. All these immunotherapeutics not only boost the immune response at the primary tumor site at the time of treatment, but also elicit systemic and prolonged protective effects by strengthening the surveillance and clearance function to prevent tumor metastasis and recurrence [82]. To date, cancer immunotherapy has been successfully applied in multiple advanced-stage malignancies; however, problems have arisen with its development. For example, immune toxicity and auto-immunity are increasingly recognized as serious clinical issues. Failure to address these issues stemming from prior failures could result in the reiteration of errors similar to those seen in some conventional therapies, thereby impeding the advancement and broader implementation of immunotherapy.

#### Cancer vaccines

Antigen-driven cancer vaccines can equilibrate the crosstalk between the tumor cells and the host immune system by enhancing pre-existing immunity. Cancer vaccines come into two types: prophylactic (preventative) and therapeutic (curative) [81]. It’s undeniable that the considerable success of prophylactic vaccines in preventing cancer of viral origin has been made. The most representative examples are hepatitis B virus (HBV) [83] and human papillomavirus (HPV) [84, 85] in preventing liver and cervical cancer. Meanwhile, the development of genomics and proteomics promotes the fast-evolving of therapeutic vaccines, making it easier to understand the nature of tumor-mediated tolerogenic and antigen presentation, and to identify viral antigens and mutated neo-antigens that are not subject to thymus-induced tolerance [86]. Although several clinical trials have been conducted to explore the anti-tumor efficacy of cancer vaccines and noticeable progressions have been made (Table 1), the widespread use of cancer vaccines is still a challenge. Reasons are included but not limited to (1) antigenic drift [86], (2) high disease burden, immune-suppressive and regulatory mechanisms undermining the efficacy of vaccines [87], and (3) suppressive immune cells, mainly including Regulatory T cells (Tregs) [88], TAMs [89] and myeloid-derived suppressor cells (MDSCs) [90, 91] that dampen the host immune responses.

**Table 1:** Clinical trial examples of current cancer immunotherapy

**Category**	**Type**	**Cancer **	**Drug Name**	**Identifier; Phase; Refs**	**First posted; ** **Status; ** **Country **	**Results**
Cancer vaccines	Cell-based vaccine	PDA	GVAX	NCT02004262;II; [92]	2018; Completed; USA	Pancreatic tumor vaccine GVAX, which is a granulocyte-macrophage colony-stimulating factor-secreting allogeneic pancreatic tumor cells, in combination with cyclophosphamide and CRS-207 (a live attenuated *Listeria monocytogene* expressing mesothelin), can improve the median OS of PDA patients, which is better than the historical OS achieved with chemotherapy.
	DC vaccine	GBM	CSC-mRNA-Based DC Vaccine	NCT03548571; II/III; [93]	2018; Completed; USA	Seven GBM patients who received the CSC-mRNA-based DC vaccine did not develop adverse autoimmune events or other side effects. Compared to matched controls, progression-free survival was 2.9 times longer in vaccinated patients.
	Microbial vector Vaccines	PC	Prostvac	NCT01322490; III; [94]	2011; Completed; USA	Microbial vector vaccine PROSTVAC, a viral vector–based immunotherapy, prolongs median OS by 8.5 months versus placebo in metastatic castration-resistant PC patients.
		Cervical cancer; HNC	ADXS-HPV	NCT01266460; II; [95]	2010; Completed; USA	Therapeutic HPV vaccine ADXS-HPV is tolerable for women with persistent, recurrent, and/or metastatic HPV-related cervical cancer.
		CRC	VB-111	NCT04166383; II; [96]	2020; Completed; USA	The combination of microbial vector vaccine VB-111 with the PD-1 monoclonal antibody nivolumab treatment cannot improve the OS of patients, but the drug has low toxicity in patients.
	mRNA vaccines	Melanoma	BNT111	NCT02410733; I	2015; Completed; Germany	mRNA vaccine BNT111 is an effective immunotherapy for melanoma patients who are tolerant to immune checkpoint inhibitors.
		Melanoma	mRNA-4157	NCT03897881; II; [97]	2019; Recruiting; USA	In high-risk melanoma patients with removed tumor tissue, the combination of the adjuvant microbial vector vaccine mRNA-4157 and PD-1 antibody pembrolizumab can prolong recurrence free survival and has controllable safety compared to pembrolizumab monotherapy.
	Exosome-based vaccine	NSCLC	IFN-γ-Dex	NCT01159288; II; [98]	2010; Competed; France	In maintenance immunotherapy for NSCLC patients with stable or responsive chemotherapy, exosome-based vaccine IFN-γ-Dex treatment can enhance the function of NKp30 dependent NK cells, but has no induction effect on antigen-specific T cell responses, making it a well-tolerated immunotherapy.
ACTs	TILs-based ACTs	Melanoma	Melanoma antigens-specific CD8^+^ T cells	NCT02424916; I/II; [99]	2015; Completed; France	Melanoma antigens-specific CD8^+^ T cells have the characteristics of safety and good immunotherapeutic effect, which can be further enhanced by the selection of highly reactive T cells.
		HPV-associated cancers	TCR- engineered T cells targeting HPV-16 E7	NCT02858310; I/II; [100]	2017; Recruiting; USA	TCR-engineered T cells targeting HPV-16 E7 for patients with metastatic HPV-associated epithelial cancers can mediate regression of common carcinomas.
	CAR-based ACTs	GBM	CAR-T cells targeting EGFR	NCT05168423; I; [101]	2023; Recruiting; USA	Preliminary safety and bioactivity of CAR-T cells targeting EGFR have been observed, and an encouraging early efficacy signal has been detected requiring confirmation with longer follow-up time in rGBM patients.
	CAR-based ACTs	DLBCL; CLL	CD19-targeted CAR T cells	(1) NCT03344705; I; [102] (2) NCT02640209; I; [103]	(1) 2017; Completed; China; (2) 2016; Completed; USA	(1) 5 patients with recurrent or refractory DLBCL received treatment with CD19-targeted CAR T cells, namely 19-41BBz-CAR-T cells. Among the 4 evaluable patients, 2 received CR, 1 received PR, and 1 had SD; (2) 19 CLL patients received CD19-targeted CAR T cells treatment, of which 18 patients developed CRS; Among the 18 subjects, 15 had grades 1-2, and 5 developed neurotoxicity. The 3-month CR rate of patients was 44%, and at 12 months, 72% of the subjects did not have MRD.
	CAR-NK-based ACTs	B-cell cancers	CD19 CAR-NK cells	NCT03056339;I/II; [104]	2017; Completed; USA	Of the 11 patients who were administrated of CAR-NK cells , 8 (73%) had a response; of these patients, 7 (4 with lymphoma and 3 with CLL) had a complete remission, and 1 had remission of the Richter’s transformation component but had persistent CLL. Responses were rapid and seen within 30 days after infusion at all dose levels. The infused CAR-NK cells expanded and persisted at low levels for at least 12 months.
ICIs	anti-PD-1 ICIs	NSCLC	MK-3475	NCT01295827; I; [105]	2011; Completed; USA	The PD-1 monoclonal antibody MK-3475 at a dose of 2 mg/kg or 10 mg/kg every 3 weeks might be an effective treatment in patients with few effective treatment options.
			Nivolumab	NCT01642004; III; [106]	2012; Completed; USA;	The anti-PD-1 monoclonal antibody Nivolumab continued to demonstrate clinically meaningful OS, PFS, and DOR benefits versus docetaxel and maintained a favorable safety profile.
		cHL	MK-3475	NCT02453594; II; [107]	2015; Completed; USA	In a subgroup of severely pretreated cHL patients, the anti-PD-1 monoclonal antibody MK-3475 blocking PD-1 has significant clinical activity and can induce high ORR in chemotherapy resistant cHL.
	anti-PD-L1 ICIs	mUC	Atezolizumab	NCT02108652; II; [108]	2014; Completed; USA	The anti-PD-L1 antibody Atezolizumab exhibits durable activity and good tolerability in the patient population, increased levels of PD-L1 expression on immune cells is associated with increased response.
mAbs	CTLA-4 targeting mAbs	aLC	Yervoy	NCT01658878; I/II; [109]	2012; Ongoing; USA	ongoing
OVs therapy	Protoparvovirus based OVs	PDAC	ParvOryx	NCT02653313; II; [110]	2015; Completed; Germany	The protoparvovirus-based OV parvOryx exhibits good tolerance without dose limiting toxicity, with 1 patient exhibiting confirmed partial reactions and another exhibits unconfirmed partial reactions. The survival time of the two patients is 326 days and 555 days, respectively.
	Vaccinia virus based OVs	Metastatic cancer;	BT-001	EudraCT202000050580; I/II; [111]	2020; Ongoing; France	ongoing
		Advanced solid tumors	ASP9801	NCT03954067; I; [112]	2019; Completed; Japan	The administration of oncolytic acne virus ASP9801 makes tumors sensitive to ICIs, and has anti-tumor activity in both direct injection and distant non injection tumors.
	Herpes simplex virus type II based OVs	Advanced solid tumors	oHSV-2	NCT05216965; I/II; [113]	2022; Recruiting; China	oHSV-2 has good safety; the highest non severe toxic dose in monkey toxicology research is 6mg/kg, with relatively mild adverse events.

*PDA* Pancreatic adenocarcinoma, *OS* Overall survival, *GBM* Glioblastoma, *CSC* Cancer stem cell, *DC *dendritic cell, *PC* Prostate cancer, *ADXS-HPV* Axalimogene filolisbac human papillomavirus, *HNC* head and neck cancer,*CRC, *Colorectal cancer, *NSCLC* Non-small cell lung cancer, *Dex* Dendritic cell-derived exosomes, *ACTs* Adoptive cell therapies, *EGFR* Epidermal growth factor receptor, *rGBM* Recurrent glioblastoma, *DLBCL* Diffuse largeB-cell lymphoma, *CLL* Chronic lymphocytic leukemia, *CR* Complete response, *PR* Partial response, *SD *Stable disease, *CRS* Cytokine release syndrome, *MRD* Measurable residual disease, *ICIs* Immune checkpoint inhibitors, *DOR* during of response, *cHL* Classical hodgkin lymphoma, *mUC* Metastatic urothelial carcinoma, *aLC* Advanced hepatocellular carcinoma, *mAbs *Monoclonal antibodies, *PDAC* Pancreatic ductal adenocarcinoma, *OVs *Oncolytic virus, *oHSV-2* Oncolytic herpes simplex virus type 2

#### ACTs

ACTs are personalized treatment strategies, and the Food and Drug Administration (FDA) has approved two types of CAR-T (CD19 CAR-T and BCMA CAR-T) cells for clinical trials [114, 115]. Besides these two, other types of immune cells also make achievements. For instance, TILs possess the advantage of being abundantly available. However, these cells often exhibit dysfunction due to their isolation from tumor tissues characterized by a high degree of non-synonymous gene mutations [116, 117]. Engineered TCR T cells have the advantage of being able to target intracellularly expressed proteins [118], but are limited by the diversity of human leukocyte antigens (HLA) present in the human population. CAR-T cells can be generated as individualized therapies in most patients but targeting is generally restricted to tumor-associated antigens that are expressed on the surface of tumor cells [119]. Recently, NK cell therapies have delivered promising results, showing encouraging efficacy and remarkable safety. Our group has conducted extensive research on NK cell-based cancer immunotherapy, which confirms that NK cell therapy has allogeneic potential and can be a powerful anti-cancer weapon [75, 120, 121]. Using a model with engrafted human hematopoietic cells (hHCs) in an immune-deficient mouse model, a previous preclinical study indicated that CD123 CAR-NK cells are safe (5-day OS: 100%) but not for CD123 CAR-T cells (5-day OS: 0%), although the two types of CAR immune cells have comparable antileukemia efficacy [122–124]. However, some challenges also exist for NK cell-based immunotherapy, such as the short half-life in vivo and limited expansion of NK cells in vitro [125]. Macrophages are one of the most promising innate immune cells, with attractive features like their phagocytic activity, capability for antigen presentation, and flexible phenotypes; however, the limited ex vivo expansion ability compared to T and NK cells and the difficulty of genomic programming with high purity are the two obstacles to overcome [126].

#### ICIs

ICIs are a class of immunotherapeutics that induce T cell-mediated anti-tumor responses by selectively blocking the inhibitory checkpoint receptors subject to manipulation by cancer cells [127]. Taking co-inhibitory receptors, CTLA-4 and PD-1/PD-L1, as an example, (1) ipilimumab, also named as Yervoy, is a monoclonal anti-CTLA-4 antibody. It was demonstrated that ipilimumab can shrink solid tumors and improve the overall survival rate of patients with advanced melanoma (stage III and IV). It has also been approved for adjuvant therapy (stage III melanoma) [109]. However, the blockade of CTLA-4 is accompanied by excessive activity of T cells, which not only enhances anti-tumor immune response but can also results in serious clinical toxic side effects, such as autoimmune diseases in the digestive system, liver, skin, and the nervous system [109]. (2) Similarly, targeting PD-1/PD-L1 can result in the overly activated immune T cells [128]. PD-1/PD-L1 related drugs are mainly proven effective in malignant solid tumors, and the overall effective rate in the non-selected population is not high. Thus, despite the success of anti-CTLA-4 and anti-PD-1/PD-L1 therapies, only a fraction of patients benefit from ICIs [129, 130].

#### MAbs

As one of the promising strategies in cancer therapeutics, the development of mAbs will continue to progress unhindered. mAbs exhibit the ability to selectively bind to antigens, inducing cytotoxicity through neutralizing or proapoptotic mechanisms, while concurrently fostering innate immune responses [131], such as antibody-dependent cellular cytotoxicity (ADCC) [132], complement-dependent cytotoxicity (CDC) [133], and antibody-dependent cellular phagocytosis (ADCP) [134]. Despite the notable clinical successes of antibody therapy, many of the mechanisms of action and their clinical relevance remain poorly understood, and therapeutic resistance remains a major challenge. Moreover, cytokine release syndrome and tumor lysis syndrome are two insurmountable hurdles [135, 136]. To better select suitable patients for mAbs therapy, a method named quantitative systems pharmacology (QSP) has been applied in a clinical trial which aimed at exploring the anti-tumor efficacy of Yervoy in treating advanced liver cancer [137].

#### OVs therapy

As an emerging facet of immunotherapy, OVs represent a category of viruses, either naturally occurring or artificially engineered, capable of selective replication within cancer cells, culminating in cell lysis [76, 138]. OVs can not only be used as oncolytic agents alone but they also can be used as effective carriers of anti-cancer genes and play multiple functions simultaneously such as virotherapy and gene therapy [139]. T-vec (Talimogene laherparepvec, Imlygic), for the topical treatment of unresectable skin, subcutaneous, and lymph node lesions in patients with recurrent melanoma was the first OV drug approved by the U.S. FDA, which was approved in 2015 [140]. Compared to traditional chemotherapy and radiotherapy, OVs lyse cancer cells, without damaging normal cells, tissues, and organs [138]. Therefore, oncolytic virotherapy shows potential to achieve efficacy for various types of cancers and improve the overall survival in cancer patients, which has been considered to be a promising anti-cancer therapy [141]. Nevertheless, oncolytic virotherapy’s effectiveness primarily relies on combination therapies, with the stand-alone efficacy of oncolytic viruses being subject to variability based on factors such as patients’ immune status, tumor type, and choice of oncolytic viruses. Additionally, the current OVs also have relatively poor tumor penetration and can be quickly cleared by anti-viral responses [142].

Overall, tumor immunotherapy strategies, such as cancer vaccines (e.g., cell-based vaccines), ACTs (e.g., CAR-based-ACTs, TCR-based-ACTs), ICIs (e.g., anti-PD-1 ICIs, anti-PD-L1 ICIs), mAbs, and OVs therapy (e.g., Vaccinia virus based OVs) have implemented clinical trials and some have achieved good results. The relevant clinical trials are shown in Table 1 [92–113]

### The era of nanomedicine

The ultimate goal of cancer immunotherapy is to prevent cancer cell metastasis and recurrence by stimulating the immune system, and finally achieve the purpose of treatment by eliminating cancer cells. Clearly, to achieve this purpose, an active immune system with a robust and controllable immune function is necessary. However, in most situations, “robust and controllable” is challenging. Insufficient immune responses and over-activated autoimmunity are the main dilemmas in current clinical immunotherapeutics. For example, cancer vaccines can induce activated immunity for only a specific type of cancer [143], ACTs can be rendered dysfunctional as a result of tumor-mediated immune suppression [144], ICIs can cause immune-related adverse organ damage [145], and mAb therapies can result in over immune response like cytokine release syndrome [146]. Additionally, pathological and physiological barriers in our body can impede the access of immunotherapeutic drugs or native and engineered immune cells to cancer tissues and cells, making the delivery of cancer immunotherapeutic “drugs” much more difficult. Moreover, some kinds of tumors respond poorly to immunotherapeutics because of lacking an immunogenic microenvironment [147, 148]. These issues hamper the development and widespread use of cancer immunotherapeutics. Lots of efforts have been made to improve cancer immunotherapy, and within those strategies, nanoparticles (NPs)-based nanomedicine seems to be an attractive platform to overcome these shortcomings.

The concept of nanomedicine emerges in 2000 for enhancing early diagnosis and treatment of diseases such as cancers, diabetes, Alzheimer’s, Parkinson’s, and cardiovascular disease [149]. Recently, NIH re-defined it as described in the Introduction part. At present, nanomedicine can be divided into two categories: one is nanoparticle medicine, the nanometerization of the medicine itself, that is, the medicine is made into nanometer size by certain methods (e.g., suspensions, tablets, capsules) [150]; the other is nanocarrier medicine, the medicine loaded into nano-vehicles, the effect of nano-vehicles can better promote drug efficacy (e.g., liposomes, nanospheres, nanocapsules) [151–153].

**Fig. 2:**
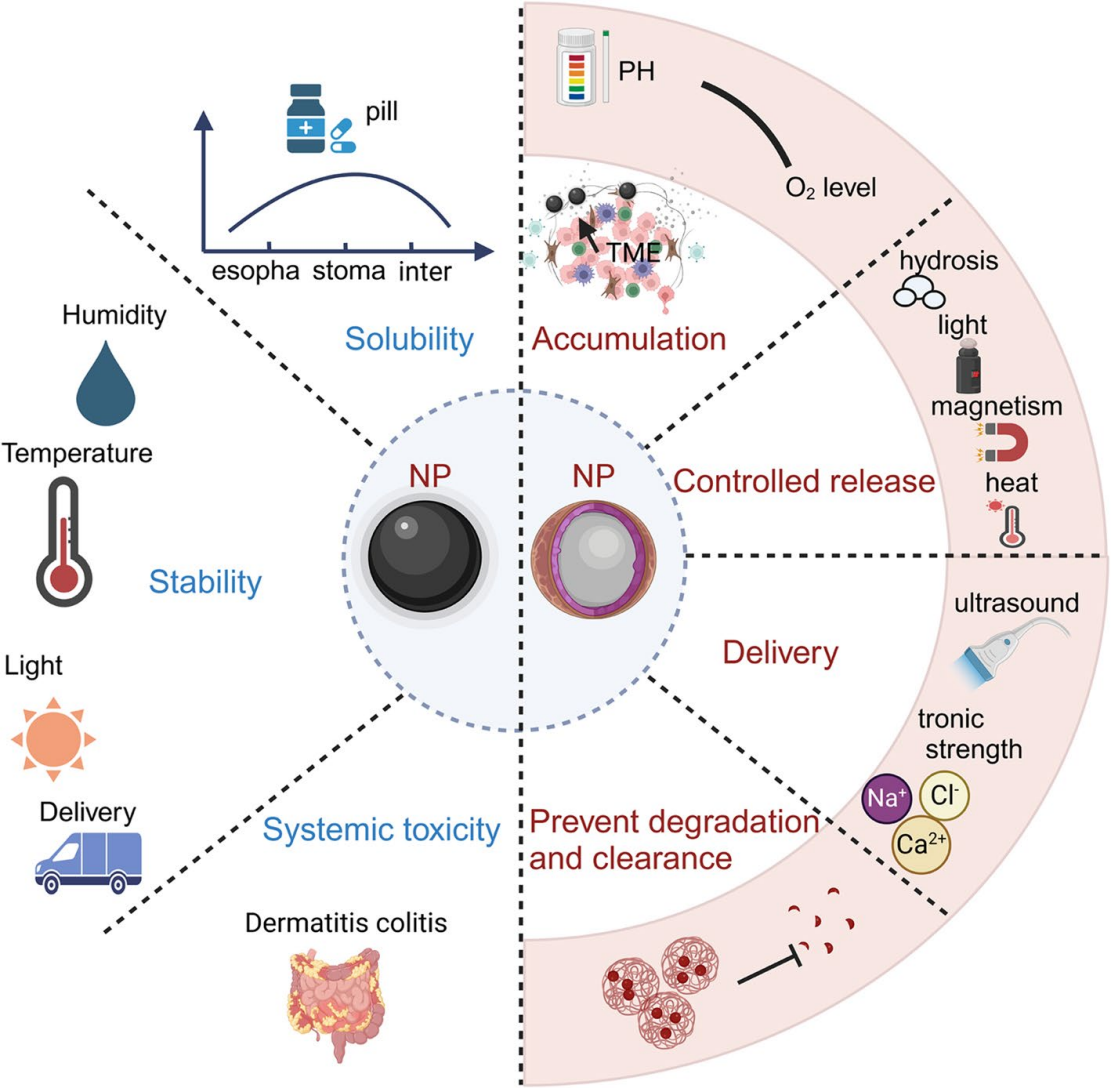
Parameters and properties of nanomedicine-based cancer immunotherapeutics. The middle circle represents two types of nanomedicine: nanoparticle medicine, which is the nanometerization of the medicine itself (black) and nanocarrier medicine, in which the medicine is loaded into nano-vehicles. The left circle is solubility, stability, and systemic toxicity, which need to be considered when mentioning “drug”. The right middle circle represents the nanomedicine can improve the accumulation of drugs in the TME, control the release of drugs, target drug delivery, and even prevent degradation and clearance of drugs during circulation after intaking. The outside right circle is the driving force of the right middle circle

**Fig. 3:**
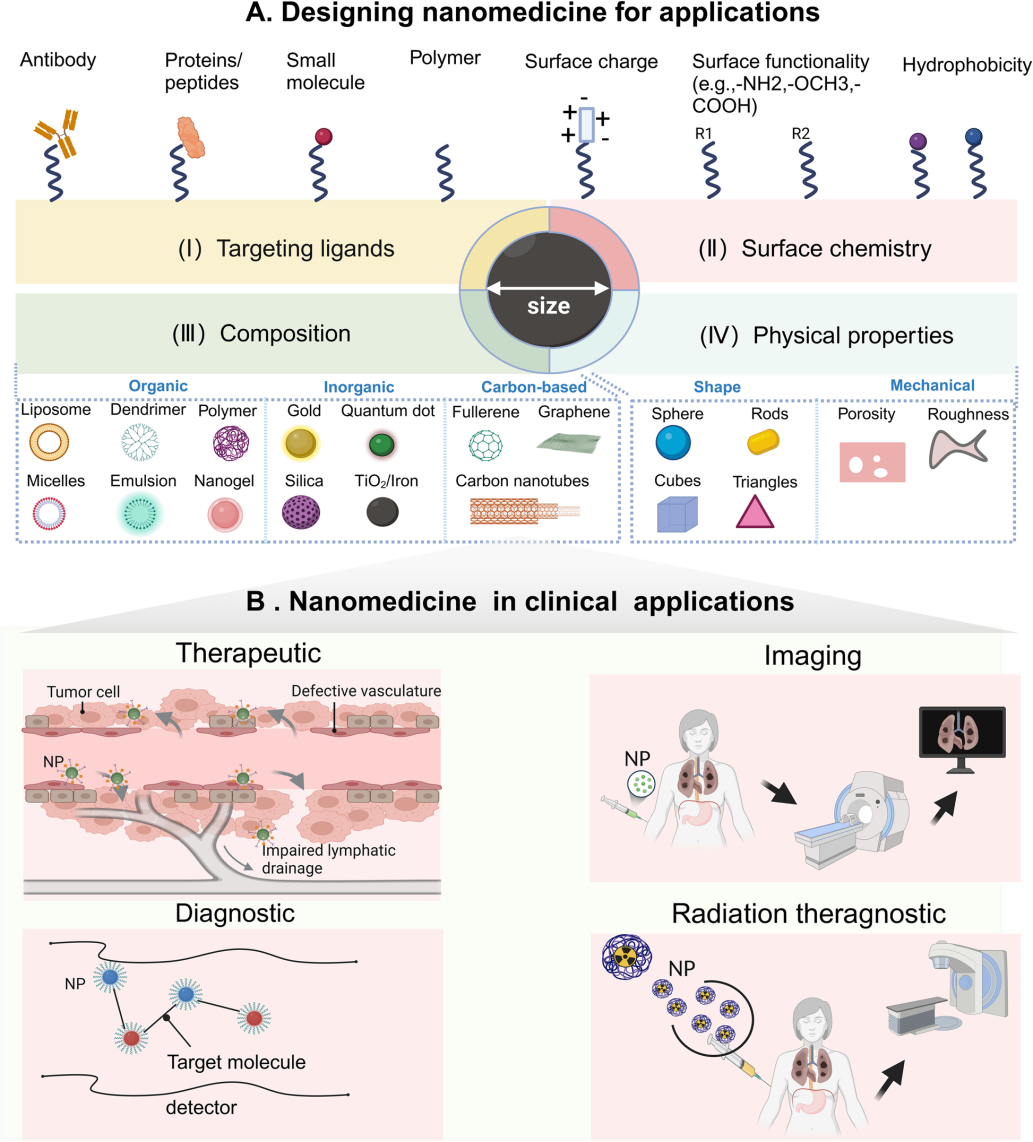
The diversity of the nanoparticle -based nanomedicine platform. Its properties are based on two points: **A.** designing nanomedicine for applications, which according to targeting ligands, surface chemistry, composition (organic, inorganic, and carbon-based), and physical properties (shape, or mechanical); **B.** nanomedicine in clinical applications (therapeutic, diagnostic, imaging, or radiation theragnostic)

As shown in Fig. 2 (left), the level of solubility, stability, and systemic toxicity are all safety indicators that should be considered for nanomedicines. Meanwhile, as demonstrated in Fig. 2 (right), some characteristics of NPs, such as ultra-small size, modifiable surface, and some special physicochemical characteristics are suitable for nanomedicine. As examples of outcomes, nanomedicine can increase the accumulation of drugs in the TME, controlled release of drugs, as well as target drug delivery, and even prevent degradation and clearance of medications in the circulation. The diversity of the nanomedicine is determined by the different types of NP which are shown in Fig. 3A: (I-II) surface modification: targeting ligands or surface chemistry; (III) composition: organic, inorganic, and carbon-based [154]; (IV) physical properties: shape or mechanical [155]. Moreover, many nanomedicines have been approved by the FDA for the corresponding clinical application [156–159]. This application field of nanomedicines mainly focuses on: therapeutic, diagnostic, imaging, or radiation theragnostic (Fig. 3 B). All types of nanomedicine platforms have made important contributions to oncology over the past several decades. For example, the biological and polymeric NPs can be adjusted as delivery tools due to their excellent biocompatibility, controlled release, and easily changed compositions and surface functions [160, 161]. PEGylated nano-liposomal Doxil® becomes the first FDA-approved nanomedicine [162]. Inorganic NPs have unique thermal, optical, magnetic, and electrical properties, and their chemical properties can include inertness, and stability, and can be easily engineered to perform specific functions, like regulating cell behaviors by regulating specific cellular signaling [163]. Inorganic and metallic nanomedicine Nanotherm® (MagForce), which allows cell uptake and introduces superparamagnetism to treat glioblastoma, was approved by the FDA in 2010 [164]. Crystalline nanomedicine takes advantage of the ultra-large surface-to-volume ratio of NPs to increase the dissolution velocity and saturation solubility. Additionally, increased saturation solubility due to the decreased nanoscale dimension results in the enhanced driving forces for diffusion-based mass transfer through biological structures (e.g. walls of the gastrointestinal tract), providing a feasible solution for the poorly soluble drugs [165, 166]. Both organic and inorganic materials can make the crystalline nanomedicine. The representative example is the first three FDA-approved nanocrystals: Rapamune®, Tricor®, and Emend®, three of which produced by an elan nano-system using milling approach. These nanomedicines are expected to be broadly used as bone graft substitutes that can overcome the solubility issues [164, 167]. The representative examples are covalent organic frameworks (COFs) and crystalline metal nanoparticles. The properties and functionalities of COFs are determined by the synthesis method, and thus, different synthesis methods can provide COFs with diverse functionalities which lead to the COFs’ widespread use [168]. For example, iodine- and ferrocene-loaded covalent organic framework (TADI-COF-Fc) can enhance radiotherapeutic efficacy in the treatment of radioresistant esophageal cancer, as iodine atoms on the COF framework can increase the production of reactive oxygen species (ROS) [169]. Crystalline gold nanoparticles (AuNPs) can induce mitochondria-mediated apoptosis and cell cycle arrest of cancer cells since they can depolarize the mitochondrial membrane of cancer cells [170].

Overall, NPs can be endowed with various functions. NPs-based nanomedicine is a good auxiliary platform to facilitate chemotherapy, radiotherapy, hyperthermia as well as cancer immunotherapy [171, 172].

## Nanomedicine platform for cancer immunotherapy

How does nanomedicine platform assist cancer immunotherapy? How does the nanomedicine platform revolute cancer immunotherapy? To answer these two questions, we need to know the nature of cancer immunotherapy. All types of cancer immunotherapies, in nature, are “drugs”. Solubility, stability, and toxicity are parameters that need to be considered when going to clinical trials. Accumulation, release, delivery, degradation, and clearance are factors that need to be considered when mentioning drug efficiency. The limitations of cancer immunotherapies are summarized in the “*Pros and cons of cancer immunotherapy*” part.

To address the limitations associated with current immunotherapy, numerous nanomedicines have been developed, leveraging their advantageous attributes for various aspects of immunotherapy, including drug delivery, stimulation of anti-tumor immunity, management of tumor immune evasion, and even synergistic administration of multiple immunotherapeutic agents [173–175]. These nanomedicines can be generated with various compositions, properties, and modified surfaces based on their treatment purpose [176]. For example, to improve the antigen uptake and presentation process, nanomedicine generated with liposome composition or surface modified with positive charge can be used to increase the efficiency of the antigen uptake process of DCs [177]. Additionally, the size of the nanomedicine plays an important role in DCs trafficking mechanisms. Manolova et al*.* found that only small-size NPs (< 200 nm) can be free drainage to lymph nodes and target lymph node-resident DCs, while larger NPs (> 500 nm) have to be incorporated by skin-resident DCs for transportation to lymph nodes [178]. Nanomedicine is an exciting platform to assist cancer therapy and cancer immunotherapies, and promote their rapid development and widespread. Different kinds of nanomedicine have been generated to specifically target either tumor cells or immune cells (e.g., HER2^+^ BC cells, DCs, TAMs). Table 2 shows the different types of preclinical nanomedicine tested for cancer therapy or cancer immunotherapy [179–184].

**Table 2:** Different kinds of preclinical nanomedicine tested for cancer therapy or cancer immunotherapy

Nanomedicine Platform	Treatment	Target cells	Results	Reference
Liposomal NP	HER2-targeted antibody	BC cells	MM-302, a HER2-targeted antibody–liposomal doxorubicin conjugate, combined with trastuzumab and cyclophosphamide has good tolerability and shows promising efficacy.	[179]
PLGA NP	Anti-CA19-9 antibody	PC cells	The combination of PTX-NP-anti-CA19-9 antibody and UMMD achieve the highest tumor inhibition rate in mouse pancreatic tumor cells.	[180]
Gold NP	Mannose	DCs	Using mannose modified gold NPs to deliver YTHDF1 siRNA to DCs, and promote DC maturation.	[181]
Iron Oxide NP	Mannose	M2-like TAMs	Mannose-iron oxide nanoparticles can specific delivery encapsulated contents into mannose receptor (CD206)-expressing M2-like TAMs.	[182]
RNA NP	α-M2pep	M2-like TAMs	Using α-M2pep modified M2NPs to deliver anti-CSF-1R into M2-like TAMs, resulted in a dramatic elimination of M2-like TAMs (52%), decreased tumor size (87%), and prolonged survival.	[183]
Carbon Nanotubes	Monoclonal antibody-Cetuximab targeting the EGFR	GBM cells	Carbon Nanotubes with a monoclonal antibody-Cetuximab targeting the EGFR can specifically deliver radioactive analogues into EGFR ^+^ GBM cells.	[184]

*NP,* Nanoparticle. *BC,* Breast cancer. *PC,* Pancreatic cancer. *PTX,* Paclitaxel. *UMMD,* Ultrasound-mediated microbubble destruction. *DCs,* Dendritic cells. *TAMs,* Tumor-associated macrophages. *α-M2pep* α-peptide (a scavenger receptor B type 1 targeting peptide) linked with M2pep (an M2 macrophage binding peptide). *M2NPs,* M2-like TAM dual-targeting nanoparticles. anti-*CSF-1R,* Anti-colony stimulating factor-1 receptor. *EGFR,* Epidermal growth factor receptor. *GBM,* Glioblastoma

### Delivery vehicle for immunological agents

Precision “drug” delivery is generally a problem faced by immunotherapies, especially vaccines, mAbs, and OVs. Due to the nature of our innate immune system, mucous membrane protection, epithelial and endothelial barriers, and the phagocytosis of the phagocytic cells, the agent delivery efficiency usually be compromised, resulting in modest or even weak treatment efficacy [185, 186]. Nanomedicine is the optimal option to facilitate immunotherapies to overcome these barriers due to their nanoscale dimension and ultra-large surface-to-volume ratio, which increases solubility and enhances bio-availability [187]. This is also known as an inherent advantage of NPs, the enhanced permeability and retention (EPR) effect. A study conducted by Huang et al. shows that ultrasmall AuNPs smaller than nanometer (nm) can penetrate and localize to cancer cells better than nanoparticles larger than 10 nm. In their study, quantitative inductively coupled plasma mass spectrometry (ICP-MS) measurement shows cells uptake more AuNPs with a 2 nm diameter per cell than AuNPs with 6 nm or 15 nm diameter [188]. The high renal clearance is the other benefit of the ultra-small size nanomedicine because it can reduce the nonspecific background uptake of NPs in major organs which improves tumor-specific imaging and potential toxicity. The polymeric NPs (< 10 nm) can improve EPR-based tumor targeting and efficient renal clearance [188]. As for the delivery vehicle, the excellent bio-compatibility performance makes the liposome to be a suitable system for drug delivery [189]. In a previous study, the doxorubicin (DOX) and DOX-loaded liposomes were used to co-culture with MCF-7 cells for 24 h separately, then the IC_50_ of DOX in each group was measured, and the result shows that the IC_50_ of DOX encapsulated into liposomes was lower than that of free DOX in direct contact with cells, which means that DOX-loaded liposomes are easier to deliver DOX into cells and may have stronger anti-tumor effects [190]. Moreover, polymer, self-assembled materials, micelle, and microneedles are also optional candidates for delivery vehicles [191]. Except for delivery, accumulation, and controlled release the cargo in the target cell/tissue are the other two auxiliary functions of the nanomedicine platform that can facilitate cancer immunotherapy [192, 193]. This is mainly due to their size difference, modified surface chemistry, and specific ligand targeting design. Notably, the inner of the tumor is a microenvironment with hypoxia, low pH, and abnormal expression of glutathione and enzymes [194, 195], nanomedicine could be designed as a parameter-driven drug, such as hypoxia-, enzyme- or pH-driven drug release[196]. It can be seen in a study exploring effective treatments for osteosarcoma. In this study, calcium phosphonate was used to design pH-responsive nanomedicine termed CpG- MTX@BSA-CaZol, and this nanomedicine tends to accumulate in a low-pH tumor-associated microenvironment instead of a normal tissue [197]. Nanomedicine platforms can further be light-triggered, electric pulse-sensitive, or magnetic field-navigated with intended external conditions based on their surface chemistry conditions as Figs. 2 and 3 indicated [196, 198, 199]. Importantly, the bio-compatibility and target group modification properties make the nanomedicine excellent in protecting the antigens from premature proteolytic degradation, facilitating antigen uptake and processing by antigen-presenting cells, and decreasing toxicity to the healthy cells [200–202].

Antigen degradation and inefficient antigen presentation are two major shortcomings of traditional cancer vaccines [203, 204]. Kranz et al*.* developed a universally applicable vaccine class for systemic DC targeting and synchronized induction of both highly potent adaptive as well as type I IFN-mediated innate immune mechanisms for cancer immunotherapy by optimizing the well-known lipid carriers – the intravenously administered RNA-lipoplexes (RNA-LPX). Interestingly, the LPX can protect RNA from extracellular ribonucleases and mediates its efficient uptake and expression of the encoded antigen by DCs and macrophages in various lymphoid compartments. Additionally, RNA-LPX can precisely and effectively target the DCs and macrophages by adjusting the net charge of the particles in the best way possible without adding molecular ligands to the particles. Strikingly, they also show that RNA-LPX encoding viral or mutant neo-antigens or endogenous self-antigens induce strong effector and memory T-cell responses, and mediate potent interferon-α (IFNα)-dependent rejection of progressive tumors [205].

Nonspecific delivery of mAb therapies has the potential to induce systemic toxicity, like cytokine release syndrome and tumor lysis syndrome [146, 206]. Zhang et al*.* prepared an electrostatically adsorbed trastuzumab (Tmab)-bearing PLGA/PEI/lipid nanoparticles (eTmab-PPLNs), this nanomedicine includes two parts: (1) The docetaxel (DTX)-loaded PLGA/PEI/lipid hydrophobic core, composed of poly (D, L-lactide-co-glycolide), PLGA; polyethyleneimine (PEI); and lipids. (2) Electrostatically adsorbed Tmab on the surface of PLGA/PEI/lipid core as a ligand, which can target human epidermal growth factor receptor 2 (HER2)-positive breast cancer cells. The results show that the eTmab-PPLNs as a type of polymeric/lipid nanomedicine, can target the delivery of the anti-cancer drug, enhance anticancer activity, especially, decrease toxicity towards healthy cells, and reduce chemotherapeutic dose required for treatment compared to the traditional DTX [207].

Similarly, intravenous delivery of OVs is promising in cancer treatment; however, fast clearance of OVs and the severe cytokine release syndrome impede its wide application. Huang et al*.* for the first time, used erythrocyte lipid hybrid membrane vesicle (erythroliposome) to fully encapsulate OVs for their intravenous delivery by adding artificial membranes to cell membranes. Their results show that the fluidity of the membranes is reduced, resulting in an enhanced shielding effect on OV antigens, and consequently, the OVs display less toxicity and slower clearance after intravenous infusion, and longer circulation time. Their results also indicate that this erythroliposome encapsulated OVs markedly enhanced oncolytic efficacy to metastatic and refractory tumors [208].

**Fig. 4:**
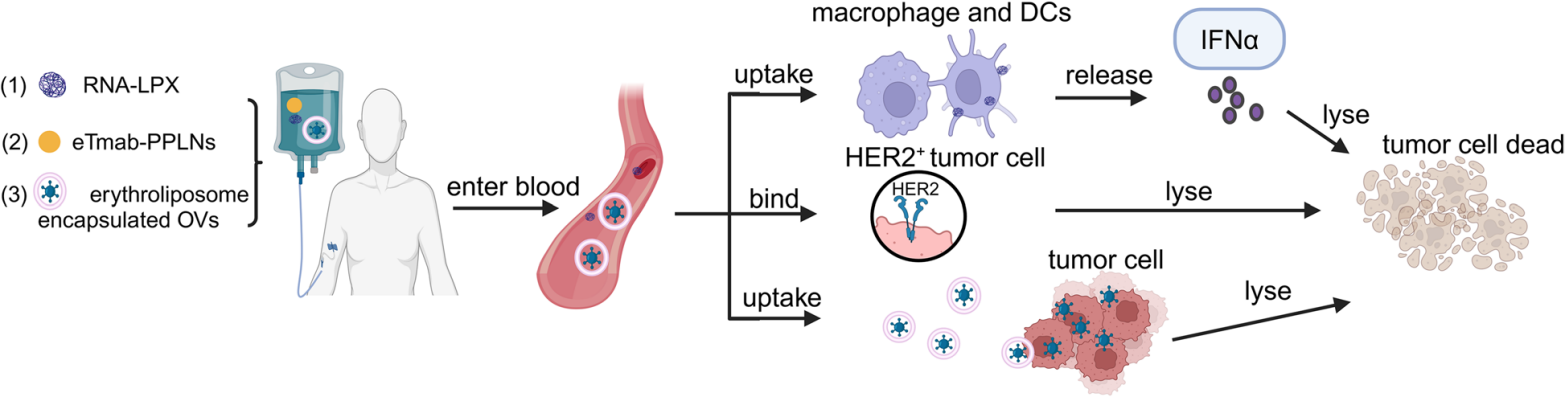
Nanomedicines act as delivery vehicles. (1) RNA-LPX can precisely and effectively target DCs and macrophages, trigger IFNα release, and induce strong T-cell responses to kill tumor cells. (2) eTmab-PPLNs target HER2^+^ breast cancer cells, transport DTX to HER2^+^ cancer cells, to lyse tumor cells. (3) Erythroliposome-encapsulated OVs are taken up by tumor cell and kill them

Overall, nanomedicine with good biocompatibility has made noteworthy contributions to drug targeting delivery and accumulation, enhanced drug stability and bioavailability, protected drugs from degradation, prolonged their half-life, importantly, and decreased their toxicity side effects [209–211]. Nanomedicine greatly compensates for the deficiency of immunotherapeutics (Fig. 4).

### Nanomedicine mediated ACT activity promotion

ACT, also known as cellular immunotherapy, is a form of treatment that uses the immune cells to eliminate cancer [49]. T cell, NK cell, macrophage cell are three representative ACTs in the current immunotherapy area [212, 213]. The future of ACT seems to be promising. However, despite these successes, challenges still remain. The high cost, time-consuming, and insufficient endogenous cell expansion due to the MHC restriction are obstacles that need to be overcome.

#### Nanomedicine in NK cell therapy

NK cell therapy recognizes their targets in an HLA-unrestricted manner and doesn’t present the risk of graft-versus-host disease (GVHD) compared to T cell [214]; however, the diversity of the NK cells and numerous regulation mechanisms, making the efficacy of NK-mediated ACT difficult to control [215]. Moreover, limited in vivo persistence and infiltration efficiency to solid tumors are other challenges to overcome [216, 217].

**Fig. 5:**
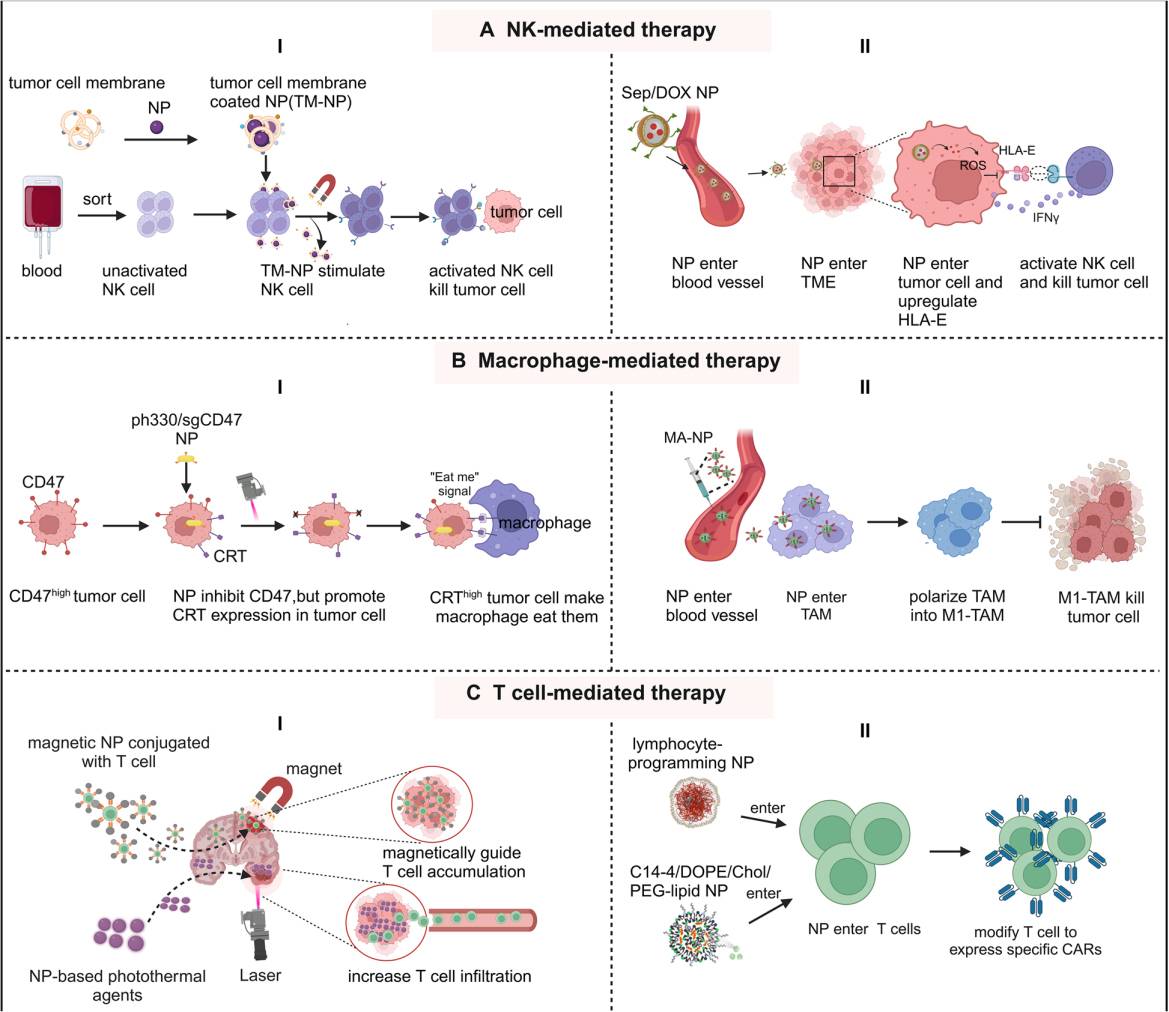
The advantages of ameliorating current NK-/Macrophage-/T-mediated therapy after integrating nanomedicine. A-I: tumor cell membrane-coated NP (TM-NP) stimulates unactivated NK cells to make the maturation and cytotoxicity of NK cells, then activated NK cells to kill tumor cells. A-II: SeP/DOX enter tumor cells in the TME, and promote the chemotherapy drug DOX accumulation and penetration in tumor cells, and then they activate NK cells by downregulating HLA-E expression to kill tumor cell. B-I: pH330/sgCD47 NP, which are PEI-coated Au nanorods with electrostatically adsorbed (CRISPR)/Cas9 plasmid pH330/sgCD47, can provide “eat me” signals to promote macrophage phagocytosis properties and increase tumor-promoting to tumor-suppressive macrophage repolarization. B-II: MA-NP targets TAM and polarizes TAM to a tumor-suppressive type, thereby inhibiting tumor progression. C-I: T cells conjugated with magnetic NPs can be easily delivered into CNS solid tumors by magnetically guiding them, and T cells combined with photothermal-mediated NPs can promote solid tumor infiltration efficiency and enhance the cytotoxicity of T cells on CNS solid tumor. C-II: lymphocyte-programming NP and C14-4/DOPE/Chol/PEG-lipid NP enter T cells and modify T cells to express specific CARs

Attractive by nanomedicine, researchers apply it into in the field of NK therapy to improve the NK cells treatment efficacy. Wu et al*.* use tumor cell membranes (TM)-coated nanomedicine to stimulate resting NK cells, and the results showed that TM-loading prompted the maturation and cytotoxicity of NK cells which potentially enhanced the effect of NK cell therapy [218]. Wei et al*.* developed a self-assembled selenopeptide nanomedicine that successfully achieves controlled transportation and synergistic responses, addressing two critical aspects when applying nanotechnology to the field of immunotherapy [219]. This system involves three parts: delivery vesicle selenopeptide (SeP), drug like DOX, and NK immune cell. Three characteristics of SeP, a tumor-targeting motif, an enzyme-responsive cleavable linker, and an alkyl chain modified selenoamino acid tail, endow SeP with advantages of enzyme-induced size-reduction and the ROS-driven deselenization. Enzyme-induced size-reduction can enhance the drug accumulation and penetration in tumor tissue, and meanwhile, the unique ROS-driven deselenization led to the downregulation of HLA-E expression in tumor cells, which could be further facilitated by DOX, suggesting the potential of on-demand activation of NK cell-mediated immunotherapy. In conclusion, the DOX-encapsulated selenopeptide nanomedicine system (SeP/DOX) can deliver therapeutics to tumor tissue, control the release of drugs, and further activate the NK cells in a programmed manner [219] (Fig. 5A).

#### Nanomedicine in macrophage-mediated immunotherapy

As for macrophages, their multiple functions (maintaining homeostasis, phagocytosis, auxiliary cells to maintain T-cell tolerance, carry out surveillance for tissue integrity, maintain tissue turnover and recruit the immune system to overcome larger tissue damage, etc*.*), wide distributions (all organs), flexible phenotypes (M1: pro-inflammatory or tumor-suppressive, M2: anti-inflammatory or tumor-promoting), and strong plasticity making them an attractive candidate for cellular immunotherapy [220]. However, their limited ex vivo expansion ability, polarized phenotype, and the lack of safe and efficient approaches for their reprogramming greatly hindered their development and application [126].

Nanomedicine holds the ability in repolarization of tumor-promoting to tumor-suppressive macrophages to effectively activate macrophages to “eat” tumor cells. Huang et al*.* generated an AuP_pH330/sgCD47_ nanocomplexes which are PEI-coated Au nanorods with an inherent capability to induce calreticulin (CRT) exposure and electrostatically adsorbed the clustered regularly interspaced short palindromic repeat (CRISPR)/Cas9 plasmid pH330/sgCD47. The AuP_pH330/sgCD47_ nanocomplexes can: (1) provide an “eat me” signal to promote the macrophage phagocytosis property, and (2) increase tumor-promoting to tumor-suppressive macrophage repolarization [221]. Ke et al*.* synthesized porous hollow iron oxide nanoparticles (PHNPs) for loading a PI3K inhibitor and further modified by mannose (MA) to target TAMs. The MA-NP showed good efficiency in targeting TAM, polarizing TAM to tumor-suppressive type, thereby inhibiting tumor progression [222]. In conclusion, the application of nanomedicine in macrophages is a possible strategy for macrophage-mediated immunotherapy (Fig. 5B).

#### Nanomedicine in adoptive T cell therapy

T cell-based ACTs, including TIL-based ACT, TCR-based ACT, and CAR-based ACT, have emerged as revolutionary immunotherapeutics for treating cancer [223]. The success of numerous clinical trials indicates its immense promise in some hematologic malignancies. However, its efficacy in solid tumors is not promising yet due to intrinsic limitations. This is exemplified by treating brain tumor in the central nervous system (CNS) with T cell therapy with the limitations of: (1) the difficulty in intertumoral delivery across anatomical niches, (2) suboptimal T cell specificity or activation, and (3) intertumoral T cell dysfunction due to the immune-suppressive TME [224].

Nanomedicine platforms may offer several advantages to overcome these limitations, as they can be designed to: (1) robustly and specifically activate or engineer T cells ex vivo, (2) encapsulate T cell-stimulating agents for positioning stimulus and controlled release, even (3) be sequentially conjugated onto T cells for added functionality without disrupting the engineering processes for TCR-/CAR-T therapies. For CNS tumors, T cells conjugated with magnetic NPs can be easily delivered into CNS solid tumors by magnetically guided. Similarly, T cells combined with photothermal-mediated NPs can promote solid tumor infiltration efficiency and enhance the cytotoxicity of T cells on CNS solid tumors [225]. Our lab conformally encapsulated donor T cells within a biocompatible and biodegradable porous film (∼450 nm in thickness) of chitosan and alginate, an approach resulting in attenuating GVHD without compromising graft-versus-leukemia (GVL) effects [226].

Genetic modification of immune cells is one of the most powerful methods to improve their tumor cell-killing efficiency, especially, CAR engineering. CD19-CAR-T and BCMA-CAR-T cell therapies are approved by the FDA for the clinical treatment of cancers [227]. CAR-viral transduction is the typical gene delivery technology with high transduction efficiency. However, the high cost, safety concerns, and small cargo capacity are the challenges that hindered its development. A nanomedicine platform can decrease the cytotoxicity and cost of the gene editing strategies by taking advantages of their composition, physical properties, as well as surface characteristics. More recently, Billingsley et al*.* designed an ex vivo mRNA delivery platform, C14-4/DOPE/Chol/PEG-lipid, by using ionizable lipid nanoparticles (LNPs) that can produce high quality of the CD19-CAR-T cells and elicit potent cancer-killing activity in Nalm-6 acute lymphoblastic leukemia cells [228]. Interestingly, Smith et al. designed an in situ CAR gene introducing NP which can program circulating T cells efficiently with a long-term lifespan. The design of the DNA-carrying NP includes: (1) The core cargo part which contains the microtubule-associated sequences (MTAS) and nuclear localization signals (NLS) modified poly (beta-amino ester) polymer and plasmid DNA encoding the leukemia-specific 194-1BBz CAR; (2) The T cell-targeting delivery shell part which is the electrostatically adsorbed polyglutamic acid to anti-CD3e f(ab’)2 fragments. This NP was demonstrated to be taken up by circulating T cells and importing their DNA cargo into the cell nucleus, and thus modify T cells to express leukemia-specific CARs. Additionally, the NP is easy to be manufactured and is very stable, which simplifies the long-term storage and reduces cost. Moreover, its clinical safety and efficiency were verified by using the sleeping beauty transposon system which is more suitable for patient usage compared with conventional lentiviral vectors [229]. All these above indicate the potential clinical utility of the NP-based nuclei acid delivery technique. Thus, nanomedicine is a highly imperative platform to revolute genetic editing technologies and expand the application of immunotherapy (Fig. 5C).

### Nanomedicine mediated ICIs therapy

As a regulatory molecule, ICPs play a pivotal role in cancer immunotherapy. ICP has intricate relationships with ICI immunotherapeutics. The essence of ICI is a branch of mAbs, specifically promoting anti-tumor T-cell responses and response factors by targeting negative regulatory proteins (ICP: CTLA-4, PD-1/PD-L1) [230]. The function of the “brake” ICP is to protect healthy tissue to avoid failure while activating lymphocytes to remove pathogens by sending T cells a series of co-stimulatory or co-inhibitory signals via receptors. Co-stimulatory and co-inhibitory signals can cause activation and functional differentiation of T cells and termination and suppression of T-cell responses, respectively. As the first-line (chemo- and radio-therapy are still mainstay) therapies for various solid and liquid tumors, three types of ICIs, CTLA-4, PD-1 interact with ligand PD-L1 and PD-L2, and anti-PD-L1 antibodies, have been approved by the FDA [231], even though the therapeutic effects are limited in most tumor patients. Based on the above description, it’s not difficult to understand that the dilemma of current ICI immunotherapeutics is that excessive ICIs will easily cause adverse side effects like cytokine release syndrome or even immune-related adverse organ damage while a single ICI and low dose levels of ICIs are not enough to reverse the tumor immune-suppressive microenvironment and control tumors completely [232, 233].

**Fig. 6:**
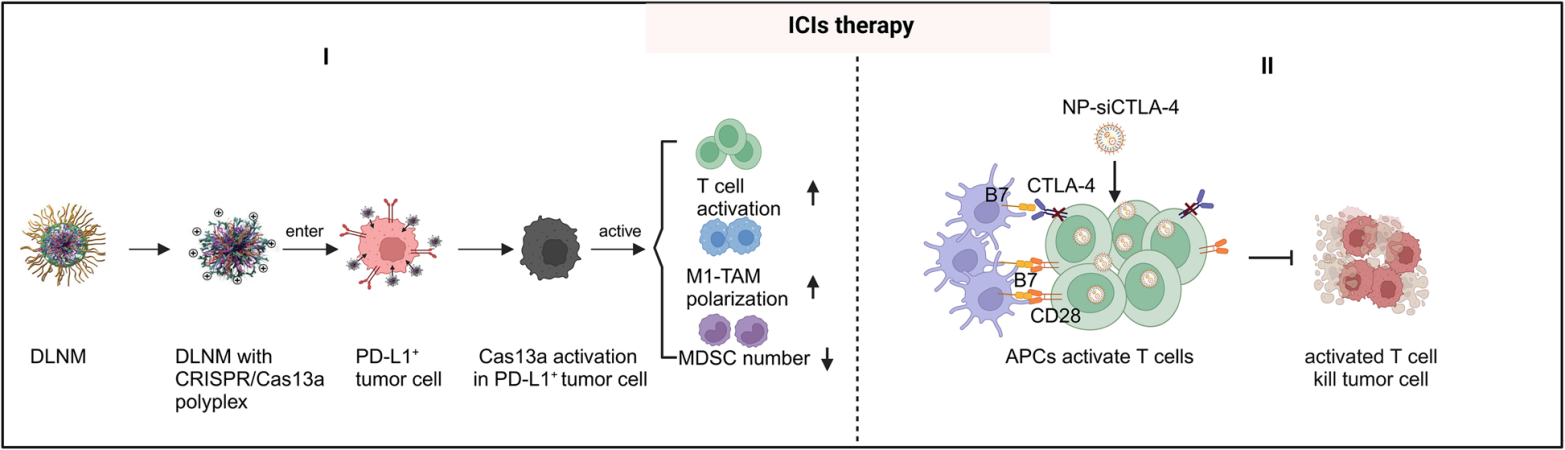
The advantages of ameliorating ICI therapy after integrating nanomedicine. I: the use of DLNM allows for targeted delivery of the CRISPR/Cas enzyme to tumor cells, enabling gene editing to disrupt PD-L1 expression and facilitate controlled release, ultimately leading to safe and effective immunotherapy activation. II: NP-siCTLA-4 delivery to T cells can inhibit CTLA-4 expression, promoting T cell activation by antigen-presenting cells (APCs) and subsequent tumor cell destruction

Applying nanomedicine is a wise option to overcome the shortcomings of the ICI therapy. These nanomedicine-ICI immunotherapeutics allow for a controllable release of ICP-related antibodies and adjuvants, and the improved tumor infiltration ability of antitumor immune cells, such as T cells [196, 234]. For example, Zhang et al*.* reported a dual-locking nanomedicine (DLNM) that can deliver the CRISPR/CRISPR-associated (CRISPR/Cas) enzyme into tumor tissues with dual stimuli response control. This DLNM can restrict the activation of CRISPR/Cas13a (Cas13a was identified as an RNA-guided RNA-targeting CRISPR effector) in tumor tissues by responding to both the microenvironment pH and the ROS concentration in the TME. Meanwhile, the dual-locking structure effectively maintains its circulation stability and prevents CRISPR/Cas13a activation by inhibiting cellular uptake of the DLNM. Once the CRISPR/Cas13a system located the tumor cell, played a role in targeting, disrupted the programmed PD-L1 through gene editing, and achieved the controlled release, thereby leading to the safe and efficient activation of T-cell-mediated immunotherapy [235]. In another study, Wang et al*.* designed a self-degradable microneedle patch to treat melanoma which can sustainedly release anti-PD-1 in a controllable manner [236]. Moreover, Li et al*.* designed a nanoparticle to deliver an ICP targeting agent, CTLA-4-siRNA (siCTLA-4), into T cells both in vivo and in vitro. The results show that siCTLA-4 was delivered into CD4^+^ and CD8^+^ T cell subsets at tumor sites. Additionally, the CD8^+^ T cell ratio is significantly increased while the percentage of the Tregs is reduced. Thus, augmented activation and anti-tumor immune responses of the TILs were achieved [237]. In summary, this nanomedicine-associated ICIs therapy strategy has the potential to achieve combination therapy for enhancing anti-tumor efficacy (Fig. 6).

#### Nanomedicine mediated TME modification

The TME is an indispensable factor that affects the outcomes of cancer immunotherapy. Dense extracellular matrix (ECM), enrichment of cancer-associated fibroblasts (CAFs), abnormal blood vasculatures, hypoxia, tumor acidosis, abundant TAMs infiltration, and tumor immune tolerance microenvironment (TIM) are seven different main characteristics compared to the normal tissue environment [238, 239]. All these common characteristics contribute to the accelerated proliferation of cancer cells, the spread of the malignant cells, and devastatingly, the destruction of the basement membrane and the promoting metastasis of cancer cells [239–241]. The TME is highly imperative in cancer progression. Reprogramming the TME as an emerging and high-impact area has made outstanding achievements in suppressing tumor proliferation and improving therapeutic effects in recent studies. Taking advantage of the inherent traits of NPs and desired properties (surface modification), an engineered drug delivery system can greatly enlarge the tumor therapeutic window by regulating the TME normalization [242].

Nanomedicine platforms developed for the above seven TME properties emerge endlessly. For example, Goodman et al*.* conjugated collagenase onto the surface of polystyrene NPs for site-specific degradation of ECM proteins, resulting in a fourfold increase in the dose of NPs entering the multicellular spheroid model [243]. In another study, Zhao et al*.* made an outstanding contribution on the pancreatic ductal adenocarcinoma (PDAC) cancer treatment by using the polymeric micelle-based nanomedicine platform (M-CPA/PTX). As one of the deadliest cancers, the excessive desmoplastic stroma makes PDAC have ineffective results with intratumoral delivery of chemotherapy medicines, meanwhile, producing a self-protection mechanism against radiotherapy. The M-CPA/PTX platform includes three parts: (1) the delivery vesicle polymeric micelle, (2) a sonic hedgehog inhibitor, cyclopamine, that can deplete CAFs, and (3) a cytotoxic chemotherapy drug, paclitaxel, that can inhibit the proliferation of the PDAC cancer. The results demonstrated that M-CPA/PTX platform could remodel the TME via depleting CAFs (28% decrease) while increase the anti-tumor effect of paclitaxel, resulting in the significant efficacy in inhabiting PDAC cancer proliferation [244]. As for paclitaxel, another attractive example is the self-assembling paclitaxel filament (PF) hydrogel which stimulated the TAMs for local treatment of recurrent glioblastoma. The novelty of this study is the “drug-delivered-by-drug” strategy, which converts the poor water solubility PTX into a molecular hydrogelator that can be used for local delivery of αCD47. Next, this aqueous PF solutions can be directly deposited into the tumor resection cavity, enabling seamless hydrogel filling of the cavity and long-acting local release, meanwhile, stimulating the macrophage-mediated immune response for local treatment of recurrent glioblastoma [245]. Overall, although challenges remain when mentioned in transfer to clinical use, using the nanomedicine platforms to make the TME have normalized immune responses is a promising strategy to broaden the tumor therapeutic window**.**

In summary, nanomedicine has great potential in the field of cancer immunotherapy. In addition to the preclinical studies mentioned in Section 4 (Figs. 4, 5, and 6; also can be seen in Table 3), several clinical studies of nanomedicine in cancer immunotherapy, such as the Lipid NP-mediated mRNA-4157 vaccine, the Lipid NP-mediated V941 drug, and MSLN-CAR T cells that secrete PD-1/CTLA-4 nanoantibodies, have been conducted, with some studies achieving good results. The relevant clinical trials are included in Table 4 [246–254].

**Table 3:** The preclinical trials of nanomedicine in cancer immunotherapy

Type	Mechanisms	Effects
Delivery vehicles	Utilizes nanoscale dimensions for enhanced solubility, bio-availability, and EPR effect	1. RNA-LPX can precisely and effectively target DCs and macrophages, trigger IFNα release, and induce strong T-cell responses to kill tumor cells. 2. eTmab-PPLNs target HER2^+^ breast cancer cells, transport DTX to HER2^+^ cancer cells, to lyse tumor cells. 3. Erythroliposome-encapsulated OVs are taken up by tumor cell and kill them.
Ameliorate current NK-/Macrophage-/T-mediated therapy	Uses nanomedicine to enhance NK cell efficacy	1. Tumor cell membrane coated NP activated NK cell to kill tumor cells. 2. Self-assembled selenopeptide nanomedicine enter TME, then enter tumor cells and upregulate HLA-E, activate NK cell to kill tumor cells.
	Nanomedicine repolarizes macrophages to enhance their anti-tumor ability	1. pH330/sgCD47 NP, which are PEI-coated Au nanorods with electrostatically adsorbed (CRISPR)/Cas9 plasmid pH330/sgCD47, can provide “eat me” signals to promote macrophage phagocytosis properties and increase tumor-promoting to tumor-suppressive macrophage repolarization. 2. MA-NP targets TAM and polarizes TAM to a tumor-suppressive type, thereby inhibiting tumor progression.
	Nanomedicine enhances T-cell delivery and functionality	1. T cells conjugated with magnetic NPs can be easily delivered into CNS solid tumors by magnetically guiding them, and T cell combined with photothermal-mediated NPs can promote solid tumor infiltration efficiency and enhance the cytotoxicity of T cells on CNS solid tumor. 2. lymphocyte-programming NP and C14-4/DOPE/Chol/PEG-lipid NP enter T cells and modify T cells to express specific CARs.
Ameliorate ICI therapy	Nanomedicine allows for controlled release and improved tumor infiltration of ICIs	1. The use of DLNM allows for targeted delivery of the CRISPR/Cas enzyme to tumor cell, enabling gene editing to disrupt PD-L1 expression and facilitate controlled release, ultimately leading to safe and effective immunotherapy activation. 2. NP-siCTLA-4 delivery to T cells can inhibit CTLA-4 expression, promote T cell activation by antigen-presenting cells and subsequent tumor cell destruction.
Mediate TME modification	Nanomedicine modifies TME to improve therapeutic effects	The polystyrene NPs with collagenase can increase the immunotherapeutic agents penetration into tumor tissues; M-CPA/PTX can remodel the TME and increase the anti-tumor effect of paclitaxel PF hydrogel can stimulate the macrophage mediated immune response for local treatment of recurrent glioblastoma.

**Table 4:** The clinical trials of nanomedicine in cancer immunotherapy

**Type**	**Cancer**	**NP-mediated drug name**	**Identifier; Phase; Refs**	**First posted; ** **Status; ** **Country **	**Effects**
Lipid NP	Solid tumors	mRNA-4157	NCT03313778;I; [246]	2017; Recruiting; USA	Lipid NP mediated mRNA-4157 vaccine encoding 20 tumor-associated antigens performs the response rate of 50% and PFS of 9.8 months in combination with anti-PD-1 antibody pembrolizumab in solid tumor patients.
Lipid NP	Advanced solid tumors; Lymphoma	mRNA-2752	NCT03739931;I; [247]	2018; Ongoing; USA	Lipid NP mediated mRNA-2752 drug encoding OX40L, IL-23, and IL-36γ activation of dendritic cells or T cells, and protection patients from tumor rechallenge.
Lipid NP	KRAS mutant Advanced NSCLC; CRC or PAAD	V941	NCT03948763;I; [248]	2019; Completed; USA	Lipid NP mediated V941 drug enhance CD8^+^T cell responses to tumor cells harboring KRAS mutations NSCLC and other kind of cancer patients.
Lipid NP	Epithelial cancer	MT-302	NCT05969041;I	2023; Recruiting; Australia	Lipid NP mediated MT-302 delivers TROP2-targeting RNA chimeric antigen receptors that express selectively within myeloid cells for in vivo immune cell programming to enhance adaptive immune response.
PLGA NP	Advanced solid tumor	PRECIOUS-01	NCT04751786;I [249]	2021; Recruiting; Netherlands	The PLGA NP mediated PRECIOUS-01 drug loads a tumor antigen of NY-ESO-1 and an iNKT cell activator IMM60 to target and augment specific antitumour immune responses in patients with NY-ESO-1-expressing advanced cancers. The results is ongoing.
Carbon NP	Advanced solid tumor	CNSI-Fe(II)	NCT06048367;I; [250]	2022; Recruiting; China	Carbon NP CNSI-Fe(II) loaded with Iron to potentiate cancer immunotherapies.
Polysiloxane Gd-Chelates-based NP	GBM	AGuIX	NCT04881032;I/II; [251]	2022; Recruiting; France	AGuIX, a nano drug based on gadolinium element, combined with temozolomide, could enhance the tumor sensitivity of radiotherapy.
PTX	Recurrent or refractory NMIBC	PPM	NCT06173349;I; [252]	2023; Recruiting; USA	Ongoing.
Polyethyleneglycolpolyethyleneimine cholesterol lipopolymer	OV	IMNN-001	NCT05739981;I/II; [253]	2023; Recruiting; USA	IMNN-001, a IL-12 DNA plasmid vector encapsulated in NP delivery system, can continuously and locally secrete IL-12 protein after cell transfection.
Nanoantibodies	Solid tumor	αPD1/CTLA-4-MSLN-CAR T	NCT06248697;I;	2023; Recruiting; China	MSLN-CAR T cells secret PD-1/CTLA-4 nanoantibody for the treatment of advanced solid tumors.
RNA-Lipoplex particle vaccine	Melanoma	RNA-LPX	NCT02410733; I; [254]	2015; Completed; Germany	The RNA-lipoplex cancer vaccine RNA-LPX can induce strong CD4^+^ and CD8^+^ T cell immunity against the vaccine antigens in melanoma patients.
RNA-lipid particle vaccines	pHGG; GBM	RNA-LP	NCT04573140;I	2021; Recruiting; USA	Ongoing.
mRNA nanocarriars	LA cSCC	mRNA-4157	NCT06295809;II/III	2024; Recruiting; USA	
mRNA nanocarriars	Melanoma	BNT111	NCT04526899;II	2021; Recruiting; USA	

*NP* Nanoparticle, *PFS *Progression-free survival, *NSCLC* Non-small cell lung cancer, *CRC *Colorectal cance, *PAAD *Pancreatic adenocarcinoma, *PLGA *Poly lactic-co-glycolic acid, *NY-ESO-1* New york esophageal squamous cell carcinoma-1, *iNKT* Invariant natural killer T cell, *PTX *Paclitaxel, *NMIBC* Non-muscle invasive bladder cancer, *PPM* PLZ4-coated paclitaxel-loaded micelle, *OV *Ovarian cancer,* MSLN-CAR *Mesothelin-targeted chimeric antigen receptor, *RNA-LPX* liposomal RNA, *pHGG *Pediatric high-grade gliomas, *GBM *Glioblastoma, *RNA-LP* RNA-lipid particle,* LA cSCC *Locally resectable advanced cutaneous squamous cell carcinoma

#### Clinical translation challenges of nanomedicine for cancer immunotherapy

Nanomedicines that incorporate some of the desired properties (e.g., conjugate antibody/protein/peptide on the surface**)** show great promise in the clinic, and more definitive results will be obtained in the near future. For example, the recombinant HER2 antigen and the AS15 adjuvant with liposome to treat metastatic breast cancer are at the Phase I/II stage [255]. However, despite the enormous progress that has been made in improving cancer immunotherapeutics by taking advantage of nanomedicine platforms, challenges are also gradually emerging and lying ahead. For example, permeability and EPR effects as foundational underpinnings for the nanomedicine platform delivery property, are thought to be the reason for nanomedicine accumulation in the solid tumor [256, 257]. Generally, most researchers believe that leaky tumor vasculature and poor lymphatic drainage are two reasons causing EPR effects [257]. However, this explanation of the EPR effect is somewhat oversimplified, as multiple biological steps in the systemic delivery of nanomedicine can influence the effect. Unfortunately, there is no clear mechanism that has been worked out yet. Moreover, little effort has been made to address the effect of EPR on nanomedicine-based cancer immunotherapy efficacy. Additionally, EPR effects in human remains largely unexplored, and our current understanding is mainly based on animal studies [258].

Another challenge that needs to be noted is the issue encountered in industrial production, where concretization involves several phases: (1) *Controllable and reproducible synthesis:* Determining the optimal physicochemical parameters like traditional drugs is essential for nanomedicine platform development. A great deal has been learned regarding independent factors such as controlled release [193], targeting delivery [259], or preventing degradation [260]. However, systematic parallel screening of numerous nanomedicine properties remains difficult due to the challenge of rapid, precise, and reproducible synthesis of nanomedicine libraries with unique features[261]. (2) *Evaluation and screening: *In vitro evaluation is important to select candidates before animal testing. However, traditional 2D culture in plate lacks the biocomplexity of natural tissues/organs and biomechanical cues like fluid flow. 3D cell culture systems (organ-on-chip or organoid) synergically work with animal models may offer a more reliable in vitro evaluation and screening system [261, 262]. (3) *Scalable manufacturing:* Good Manufacturing Practice (GMP) is a standard used to ensure that pharmaceutical products consistently meet predetermined quality standards. Manufacturing nanomedicine for the transition of it from preclinical to clinical development, subsequent commercialization, and beyond, especially the large and complex nanomedicine [258, 263, 264]. (4) *Concerns of safety:* Safety concerns should always be our priority, especially for clinical used “drugs”. The immunogenicity of both inorganic and organic nanomaterials is of concern, as these materials can interact with the host’s immune system, potentially contributing to autoimmunity or allergic reactions [265]. Also, the normal functions of nanomaterials depend heavily on their physicochemical properties, which can change significantly after they are administered and interact with biological components [266]. Thus, it can be difficult to predict in vivo performance by in vitro studies, and thus translating nanomedicine into clinical use is not straightforward. Therefore, how to introduce innovative methods to solve the above problems in the production of nanomedicines and how to improve the permeability and EPR effect of nanomedicines are challenges but provide us important opportunities in the clinical translation of nanomedicine for cancer immunotherapy.

#### Conclusions and Perspectives

We are witnessing a milestone inflection point in the field of cancer nanomedicine-assisted cancer immunotherapy. However, our current knowledge and research efforts in these two therapeutics are far from realizing the full potential of this combination modality. To facilitate the clinical development and application of cancer immunotherapeutics integrated with the platform of cancer nanomedicine, it is necessary to have a full understanding of how NPs interact with the immune system, the mechanistic insights into how NPs enable precise delivery and controlled release, and how NPs accumulate in the TME and enhance the immune response, among other factors. Controllability, reproducibility, and scalability are persistent challenges that any new generation of cancer treatment must confront. We need to determine whether nanomedicine itself possesses the necessary attributes for controllability, reproducibility, and scalability. Additionally, we must address whether nanomedicine can effectively contribute to overcoming these three “obstacles” in the clinical translation of immunotherapy. It’s important to approach these issues with objectivity.

In conclusion, we are rapidly gaining a much deeper insight into the challenges and opportunities presented by the combination of cancer nanomedicine and immunotherapy. This review has investigated the combinatory and complementary functions of cancer nanomedicine and immunotherapy that can promote the development and clinical translation of cancer therapeutics. We anticipate that this combination will shift the paradigm of cancer treatment.

## Data Availability

No datasets were generated or analysed during the current study.

## Abbreviations

DCs: Dendritic cells
NK cells: Natural killer cells
TME: Tumor microenvironment
Ags: Antigens
NIH: National Institutes of Health
ACTs: Adoptive cell therapies
TAMs: Tumor-associated macrophages
TILs: Tumor-infiltrating lymphocytes
TCR: T-cell receptor
MHC: Major histocompatibility complex
CAR: Chimeric antigen receptor
ICIs: Immune checkpoint inhibitors
PD-L1: Programmed death-ligand 1
PD-1: Programmed cell death protein 1
CTLA-4: Cytotoxic T-lymphocyte-associated antigen 4
LAG-3: Lymphocyte activation gene 3
TIM-3: T-cell immunoglobulin mucin-3
ICPs: Immune checkpoints
mAbs: Monoclonal antibodies
ADCs: Antibody–drug conjugates
cNHL: Canine non-Hodgkin lymphoma
OVs: Oncolytic virus
HBV: Hepatitis B virus
HPV: Human papillomavirus
Tregs: Regulatory T cells
MDSCs: Myeloid-derived suppressor cells
FDA: Food and Drug Administration
HLA: Human leukocyte antigens
hHCs: Human hematopoietic cells
ADCC: Antibody-dependent cellular cytotoxicity
CDC: Complement-dependent cytotoxicity
ADCP: Antibody-dependent cellular phagocytosis
QSP: Quantitative systems pharmacology
T-vec: Talimogene laherparepvec
NPs: Nanoparticles
COFs: Covalent organic frameworks
ROS: Reactive oxygen species
AuNPs: Gold nanoparticles
EPR: Enhanced permeability and retention
ICP-MS: Inductively coupled plasma mass spectrometry
DOX: Doxorubicin
RNA-LPX: RNA-lipoplexes
IFNα: Interferon-α
eTmab-PPLNs: Electrostatically adsorbed Tmab-bearing PLGA/PEI/lipid nanoparticles
DTX: Docetaxel
PEI: Polyethylenimine
HER2: Human epidermal growth factor receptor 2
GVHD: Graft-versus-host disease
TM: Tumor cell membranes
SeP: Selenopeptide
SeP/DOX: DOX-encapsulated selenopeptide nanomedicine system
CRT: Calreticulin
CRISPR: Clustered regularly inter-spaced short palindromic repeat
PHNPs: Porous hollow iron oxide nanoparticles
MA: Mannose
CNS: Central nervous system
GVL: Graft-versus-leukemia
LNPs: Lipid nanoparticles
MTAS: Microtubule-associated sequences
NLS: Nuclear localization signals
DLNM: Dual-locking nanomedicine
siCTLA-4: CTLA-4-siRNA
ECM: Extracellular matrix
CAFs: Cancer-associated fibroblasts
TIM: Tumor immune tolerance microenvironment
PDAC: Pancreatic ductal adenocarcinoma
M-CPA/PTX: Micelle-based nanomedicine platform
PF: Paclitaxel filament
GMP: Good manufacturing practice
